# Trade as a structural driver of dietary risk factors for noncommunicable diseases in the Pacific: an analysis of household income and expenditure survey data

**DOI:** 10.1186/1744-8603-10-48

**Published:** 2014-06-13

**Authors:** Michelle Sahal Estimé, Brian Lutz, Ferdinand Strobel

**Affiliations:** 1 United Nations Development Programme, 304 east 45th street, FF-1176, New York, NY 10017, USA; 2 United Nations Development Programme Pacific Centre, Kadavu House 414 Victoria Parade, PMB Suva, Fiji Islands

**Keywords:** Noncommunicable diseases, Obesity, Trade, Household consumption/expenditure surveys, Pacific Islands

## Abstract

**Background:**

Noncommunicable diseases are a health and development challenge. Pacific Island countries are heavily affected by NCDs, with diabetes and obesity rates among the highest in the world. Trade is one of multiple structural drivers of NCDs in the Pacific, but country-level data linking trade, diets and NCD risk factors are scarce. We attempted to illustrate these links in five countries. The study had three objectives: generate cross-country profiles of food consumption and expenditure patterns; highlight the main ‘unhealthy’ food imports in each country to inform targeted policymaking; and demonstrate the potential of HCES data to analyze links between trade, diets and NCD risk factors, such as obesity.

**Methods:**

We used two types of data: obesity rates as reported by WHO and aggregated household-level food expenditure and consumption from Household Income and Expenditure Survey reports. We classified foods in HIES data into four categories: imported/local, ‘unhealthy’/’healthy’, nontraditional/traditional, processed/unprocessed. We generated cross-country profiles and cross-country regressions to examine the relationships between imported foods and unhealthy foods, and between imported foods and obesity.

**Results:**

Expenditure on imported foods was considerable in all countries but varied across countries, with highest values in Kiribati (53%) and Tonga (52%) and lowest values in Solomon Islands and Vanuatu (30%). Rice and sugar accounted for significant amounts of imported foods in terms of expenditure and calories, ranking among the top 3 foods in most countries. We found significant or near-significant associations in expenditure and caloric intake between ‘unhealthy’ and imported foods as well as between imported foods and obesity, though inferences based on these associations should be made carefully due to data constraints.

**Conclusions:**

While additional research is needed, this study supports previous findings on trade as a structural driver of NCD risk and identifies the top imported foods that could serve as policy targets. Moreover, this analysis is proof-of-concept that the methodology is a cost-effective way for countries to use existing data to generate policy-relevant evidence on links between trade and NCDs. We believe that the methodology is replicable to other countries globally. A user-friendly Excel tool is available upon request to assist such analyses.

## Background

Noncommunicable diseases (NCDs) are a global health and development challenge, representing the single greatest component of global mortality [1]. Low- and middle-income countries, where nearly 80% of NCD-related deaths occur, bear the brunt of this burden [1]. The situation is expected to worsen. The World Health Organization (WHO) estimates that NCD mortality will increase by 15% globally and by 20% in poorer countries in Africa, the Eastern Mediterranean and South-East Asia [1].

NCDs pose a tremendous challenge to Pacific Island countries (PICs). In 2008, NCDs accounted for roughly 60-77% of total deaths in PICs [2]. The region has some of the highest prevalences of diabetes (47%) and obesity (75%) in the world [3]. The NCD epidemic is a relatively new phenomenon in PICs. Obesity in urban areas was first reported in Samoa, Tonga and Vanuatu from 1953. Between 1972 and 1998, the mean birth weight of Tongan infants increased by 300 grams (8.8%) [4]. Between 1980 and 2008, the Body Mass Index of females in nine Oceania countries increased by more than 4 times the global average (more than 2.0 kg/m^2^ per decade compared to 0.5 kg/m^2^ per decade globally) [5]. Obesity is generally more prevalent in urban than rural areas, but these differences are rapidly diminishing [4].

While changing diets is not the sole explanation for the rise of obesity and NCDs in the region, dietary shifts are considered a major factor. Diets in PICs have undergone a major transformation in recent history, with energy-dense, nutrient-poor processed foods having largely replaced traditional whole foods. A comprehensive review of dietary studies over time shows how food patterns have rapidly evolved from traditional low-fat diets–typically based on locally produced complex carbohydrates, fish, fresh meat and leafy greens – towards increased consumption of imported refined starch, oils, fatty processed meats and fish (tinned), sugar and confectionery [4,6]. Often referred to as the ‘nutrition transition’, this gradual process was significantly influenced by colonization and World War II, which opened up transportation and trade routes to the Pacific Islands and facilitated the increased availability of imported foods [4,6]. Dietary shifts accelerated since the 1960s, particularly in urban populations, and are characterized by large increases in fat consumption. Food supply data show that total available energy and fat supply has increased in all countries by as much as 64% since 1965 [4].

Dietary changes have multiple, related causes, such as increases in wealth, social change linked to urbanization, foreign direct investment and greater economic and trade integration. The region’s diets and standards of living are closely linked to the economic conditions and policy choices of trading partners [7]. The importance of trade in the region cannot be overemphasized. Imported goods and services as a share of GDP are nearly twice as high in PICs as the rest of the world (59% in the Pacific versus 30% globally) [8]. The main regional trade framework is the Pacific Island Countries Agreement (PICTA) in which most PICs are either already active or engaged in preparations for implementation. Six countries are also members of the WTO [9]. New agreements are being negotiated: PACER Plus with Australia and New Zealand and Economic Partnership Agreements with the European Union [10].

The importance of trade to NCDs and health generally has been increasingly recognized by both the World Health Assembly and regional stakeholders [11]. PICs’ trade policies since the 1960s are considered to have had a major precipitating effect on the nutrition transition by increasing the availability of imported and increasingly processed foods [4,6,7,11,12]. Consumption of these foods has followed in line with their increased availability and the monetization of island economies. A review of dietary studies and food data for WHO indicates that the largest single increase in availability among imported processed foods since 1965 is imported vegetable oils (palm oil in particular) [4]. The review also noted that imported fat has been added to and not replaced existing traditional fat sources. The impact of trade on diets has particularly intensified since the mid-1990s when trade and investment liberalization accelerated the penetration of transnational food companies [6,11,12]. As a result of these changes, cereal products that are not produced locally (e.g., polished rice and bleached flour) have become the largest sources of energy, deepening both dietary transformations in and food dependency of PICs [4,6,7]. In light of this, a workshop on trade, trade agreements and NCDs in PICs was held in Fiji in February 2013. Jointly hosted by WHO, the Secretariat of the Pacific Community, the Pacific Research Centre for the Prevention of Obesity and Non-communicable Diseases and the UN Development Programme, the workshop brought together representatives of trade ministries, health ministries and civil society from participating countries to formulate national prioritized plans of action that could be supported by a regional joint programme [11].

In order to understand the relationship between trade and NCDs at national-level and to develop appropriate, targeted policies in response, a strong evidence base is required. The evidence has to be broad and integrative, covering and linking epidemiological outcome and risk factor data with trade and consumption data. Although tools, such as the WHO recommended STEPWise approach to surveillance (STEPs) of NCDs, exist to track NCD epidemics, implementation of STEPs can be challenging. While STEPs is intended to be implemented every 2-3 years, only 19 countries globally had conducted more than one STEPs survey by 2011, indicating that implementation of STEPs is relatively infrequent [13]. The frequency of STEPs implementation in PICS is even lower than it is globally. Fifteen countries in the South Pacific region have published results of either subnational or national STEPs surveys between the years 2002 and 2011 [14]. None of the countries in the region has conducted more than one national survey. Only one country has completed two subnational surveys. In addition, to our knowledge, STEPs data have not been used to link NCD risk factor data to trade or other structural drivers of NCDs.

National-level food data, such as FAO’s Food Balance Sheets (FBS) and individual level food consumption data, can be useful sources of information for nutrition policy development. FBS data are collected regularly, including in the Pacific Island region. However, FBS only collects national-level data, thus obscuring distributional issues within countries [15]. According to nutritionists, the most accurate data on individual food consumption can be obtained through repeat 24-hour recall and observed-weighed food record data, but these approaches can be particularly challenging to implement reliably in low- and middle-income settings [16]. They are costly and thus seldom implemented in PICs.

To help address some of these challenges and generate additional evidence for cross-sector policymaking in the Pacific, this paper reports the results of secondary analyses of existing Household Consumption and Expenditure Survey (HCES) data (e.g. Household Income and Expenditure Surveys (HIES), Household Budget Surveys (HBS), Living Standard Measurement Surveys (LSMS) etc.). Given their granularity, HCES data can be a useful complement to STEPs and national-level food data [17]. Using HCES data from five PICs, this study had the following objectives: (1) generate cross-country profiles of food consumption and expenditure patterns; (2) highlight the main ‘unhealthy’ food imports in each country to inform targeted policymaking and (3) demonstrate the potential of HCES data to analyze the links between trade, diets and NCD risk factors, such as obesity. The paper concludes with a discussion of the results, policy implications, and the broader application of the methodology as a complement to conventional food and NCD risk factor measurement instruments.

## Methods

We conducted a secondary analysis of existing data to examine links between trade, diets and NCDs. We used two main sources of data: epidemiological data that provided obesity rates and any HCES data (e.g. HIES, HBS, LSMS etc.) that included food expenditure and caloric intake information at a fairly granular level. One of the benefits of such secondary data analysis is its efficiency. HCES data, for example, are regularly collected as part of poverty surveys, so the time and expense of designing and implementing new surveys is minimized if not avoided altogether. HCES data are also fairly valid. Previous studies have found that household level expenditure data approximate data acquired from 24-hour recall surveys, making the use of expenditure data a reasonable proxy for food consumption [15,18-20].

We used obesity as an indicator for NCDs. Obesity is more closely linked to diets than using actual NCD outcome data, which incorporates many other non-dietary risk factors. With obesity being a major issue in PICs, it was a logical indicator for NCD risk. Moreover, obesity data are readily available globally, which facilitates replication of this methodology. We used country-level obesity prevalence as reported in the 2011 WHO NCD country profiles [1].

The inclusion criteria of countries in this study were the following: (1) participation in the workshop on trade, trade agreements and NCDs in PICs that was held in Fiji In February 2013, and (2) available granular dietary data. We used HCES data to extract granular level dietary data. We identified HCES in the countries studied that included the diet component, all of which were HIES. Food expenditure data were extracted from summary analyses of HIES for Samoa [21], Solomon Islands [22], Tonga [23], Vanuatu [24] and Kiribati [25]. Estimates of caloric intake were extracted from the summary analyses for Kiribati, Vanuatu and Solomon Islands. The surveys’ original implementation occurred between 2005 and 2010. They were the only such dietary data collected across the countries during this period. Other dietary data collected in the countries included STEPs and FBS, but these data were not sufficiently granular for the objectives of this study [14,17].

Detailed data collection methods for the HIES are described in the respective countries’ HIES reports and only briefly described here [21-25]. Data were collected in one to four rounds of household surveys. Sample sizes varied from 1161 to 3822 households. A stratified probability proportional to size (PPS) sample selection methodology was used based on national enumeration areas to ensure that the sample frames were representative of the entire population. The surveys collected information on household income and expenditure as well as on household demographics, employment, education attainment, and other characteristics, including access to water and sanitation and energy utilization for cooking and lighting. Individual household level data were aggregated by the respective countries’ statistics bureaus and results were presented by separating rural and urban areas and as country level data for some countries. None of the documents included alcohol or tobacco in the food expenditure data, and the majority excluded food consumed outside the home. (Table 1) Solomon Islands’ expenditure and calorie data included two individual foods that were prepared outside the home whereas the Tongan analysis included expenditure from all restaurants and cafes. To ensure consistency across countries, expenditures on restaurant and café food in Tonga were excluded from the analysis.

**Table 1:** Comparison of HIES data sources

**Country**	**Type of data**		**Type of expenditure data**		**Population included**		**Included in food expenditure and caloric data**	**Sample size**	**Year**
	**Expenditure**	**Caloric estimates**	**Price**	**Percentage of expenditure**	**Lowest 3 expenditure deciles**	**Whole population**	**Restaurants**	**Number of households**	
Samoa [21]	✓		✓		✓			2012	2008
Solomon Islands [22]	✓	✓	✓	✓	✓		✓	3822	2005/2006
Tonga [23]	✓		✓	✓		✓	✓	1640	2009
Vanuatu [24]	✓	✓	✓		✓			n/a	2010
Kiribati [25]	✓	✓	✓	✓	✓			1161	2006

The HCES data presented food expenditure either as percentage spent on individual food items or as price information for individual items. The data for Solomon Islands, Vanuatu and Kiribati were presented in per capita adult equivalents (pcae) for the lowest three expenditure deciles. The data for Samoa were provided as per capita, and the data for Tonga were presented per household. Per capita adult equivalents are acquired from “equivalence factors” where children younger than 15 years are counted as half an adult, and therefore a household with two adults and two children would equal three adult equivalents. This methodology is used to account for the downward bias that would occur in households with multiple children. All food expenditure data used in the report were based on expenditure diaries. The five countries were compared by converting all information to percentages of total food expenditure. We calculated weighted country-level averages for Solomon Islands and Vanuatu based on the proportion of the overall population sampled households represented. The summary data for Kiribati, Samoa and Tonga already provided weighted country averages.

Individual food items were classified in Microsoft Excel into one of four, not mutually exclusive food categories, namely imported/local (or both), ‘unhealthy’/’healthy’ (or both), nontraditional/traditional (or both), and processed/unprocessed (or both). The classification of foods into the traditional/nontraditional and the processed/unprocessed categories were based on previous publications examining the nutrition transition after World War II in the region [4,6,7,12,26]. Food items such as root crops, tubers, fruits, leafy vegetables and fish among others were classified as traditional, whereas items introduced to the region after the nutrition transition were classified as non-traditional. Categorization of processed/unprocessed was modified from Monteiro et al. who classified foods into three categories (unprocessed or minimally processed foods, processed culinary or food industry ingredients and ultra-processed food products) [27]. For the purpose of this study, knowing the level of processing of each food item was unnecessary, and category one in the Monteiro et al. article was renamed as unprocessed while categories two and three were merged into processed foods in this categorization. The surveys themselves do not indicate whether food items were bought or whether the items were local/imported. Moreover, the classification of imported/local was based on a discussion with an expert in the region^a^ indicating that all processed foods in the five countries were imported. Online resources were also consulted [28-31].

**Figure 1:**
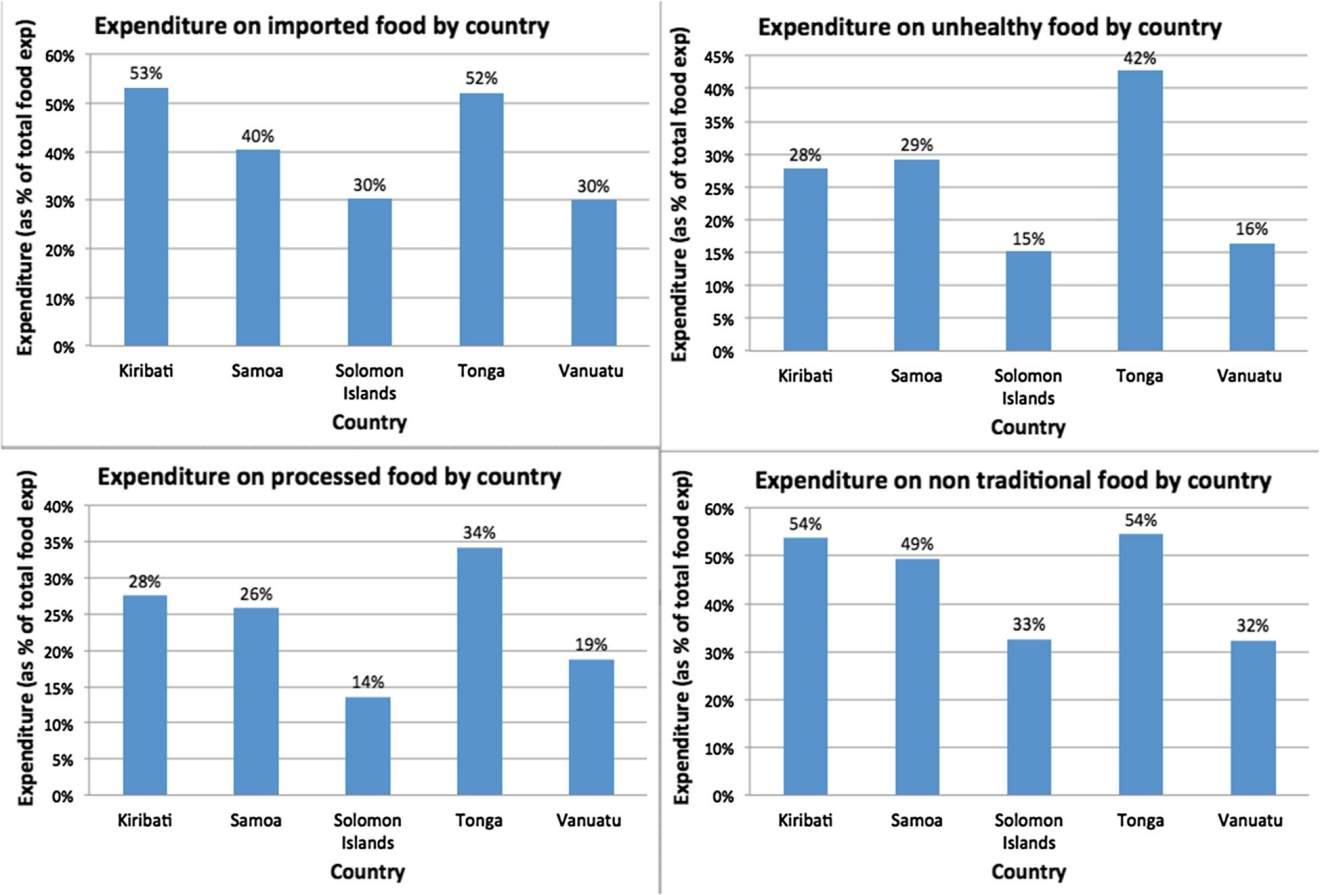
Food expenditure profiles by country.

Classifying food items into ‘healthy’/’unhealthy’ proved to be more difficult as no single definition of ‘healthy’ and ‘unhealthy’ foods exists. In fact, diets are best described as healthy or unhealthy when looking at overall food consumption rather than at individual foods in isolation, as well as in the context of physical activity and nutritional needs. Moreover, individual foods could be ‘healthy’ on some metrics like being low in saturated fats but ‘unhealthy’ on others, like high in sodium or sugars. The healthy or unhealthy food categorization was made with support from the different classifications presented in a review of existing definitions of ‘healthy’ and ‘unhealthy’, produced by Hawkes for the Canadian Office of Nutrition Policy and Promotion [32]. The review included multiple definitions of ‘healthy’ from around the world and therefore provided a comprehensive reference for this work. We categorized fruits and vegetables as healthy and other items were classified based on the classifications presented in the review where available. If an item was classified as healthy or unhealthy in a majority of classifications presented by Hawkes, the item was categorized similarly as healthy or unhealthy. If the classifications included in the Hawkes report classified a food item as healthy only if certain guidelines were fulfilled, we categorized that item in our analysis as both healthy and unhealthy. Bread is one of the items with such criteria, as several countries only classified bread as healthy if a certain percentage of the bread was made with whole wheat. In addition to using the review produced for the Canadian Office of Nutrition Policy and Promotion, context specific issues were taken into consideration. For example chicken was classified as both healthy and unhealthy as much of the chicken consumed in PICs is high in fat [4].A percentage of a particular food item’s expenditure and calorie intake was allocated to each of the four food categories (i.e., if an entire food item was considered ‘unhealthy’, then 100% of its expenditure and calorie data was allocated to the ‘unhealthy’ category). The food expenditure profiles for the five countries were then created by comparing food expenditure on each of the four food categories (Figure 1). Since the survey reports for Solomon Islands, Vanuatu and Kiribati included data on caloric intake for each food item, the same analysis was done using percentages of daily kcal pcae intakes for the three countries to generate caloric intake profiles. To allow flexibility in the categorization of individual food items or groups, percentages for the items were allocated based on the degree of spending and/or caloric intake that was imported, ‘unhealthy’, processed or nontraditional. The data used for the classification here were averages, and without a sense of the dispersion around the mean, it may be difficult to determine if certain levels of consumption are ‘unhealthy.’ Bread may be an illustrative example of this constraint in the data and in the classification scheme. Some breads may be healthier than others (especially when prepared differently in households), and some households may consume so much bread that it becomes part of an overall unbalanced diet. For ease, ambiguous food items and groups that were classified as both ‘healthy and unhealthy’ were allocated 50% of expenditure and caloric intake to the categories in question. In the case of bread, for example, 50% of expenditure and caloric intake was classified as ‘unhealthy’.

We generally did not conduct statistical analyses of the results, as the available data in the country HIES reports were already aggregated. Without access to the underlying surveys, the calculation of confidence intervals, for example, was not possible. We used Microsoft Excel, however, to conduct simple cross-country regressions to examine the expenditure and caloric intake relationships between (1) imported and ‘unhealthy’ foods and (2) imported foods and obesity rates. Though the number of data points is severely limited in the cross-country regressions, these analyses are useful proof-of-concept that the data can be used analytically. We generated pivot tables in Microsoft Excel to highlight the top imported food items in each country. We also examined the geographic internal distribution of caloric intake from imported foods in Solomon Islands, Kiribati and Vanuatu.

## Results

### Food expenditure profiles - Samoa, Solomon Islands, Tonga, Vanuatu and Kiribati

Food expenditure patterns differed among the five countries for each categorization (Figure 1). Some clustering was evident, with Solomon Islands and Vanuatu having significantly lower shares in each category. Key results are as follows:

•Expenditure on imported foods was significant in all countries, but varied considerably across countries, with the highest values in Kiribati (53%) and Tonga (52%), and the lowest values in Solomon Islands and Vanuatu (30%).

•Expenditure on ‘unhealthy’ foods was highest in Tonga at 42% of food expenditure being spent on this category and lowest in Solomon Islands with only 15% of food expenditure being allocated to ‘unhealthy’ foods.

•Expenditure on non traditional foods was similar in Kiribati, Samoa and Tonga, where 54%, 50% and 54%, respectively, was spent on this category.

•Tonga also spent the largest percentage on processed foods (34%) whereas Solomon Islands spent the least on this category (14%).While households in all the countries spent less on processed and ‘unhealthy’ foods than imported and non traditional foods, processed and ‘unhealthy’ foods still represented sizeable portions of household food expenditure. Exceptions were Solomon Islands and Vanuatu, where expenditure on these foods was as low as 15% and 16%, respectively, for ‘unhealthy’ foods (Figure 1). Households in Tonga generally spent most across all categories, whereas households in Solomon Islands generally spent the least.

### Caloric intake profiles - Solomon Islands, Vanuatu and Kiribati

**Figure 2:**
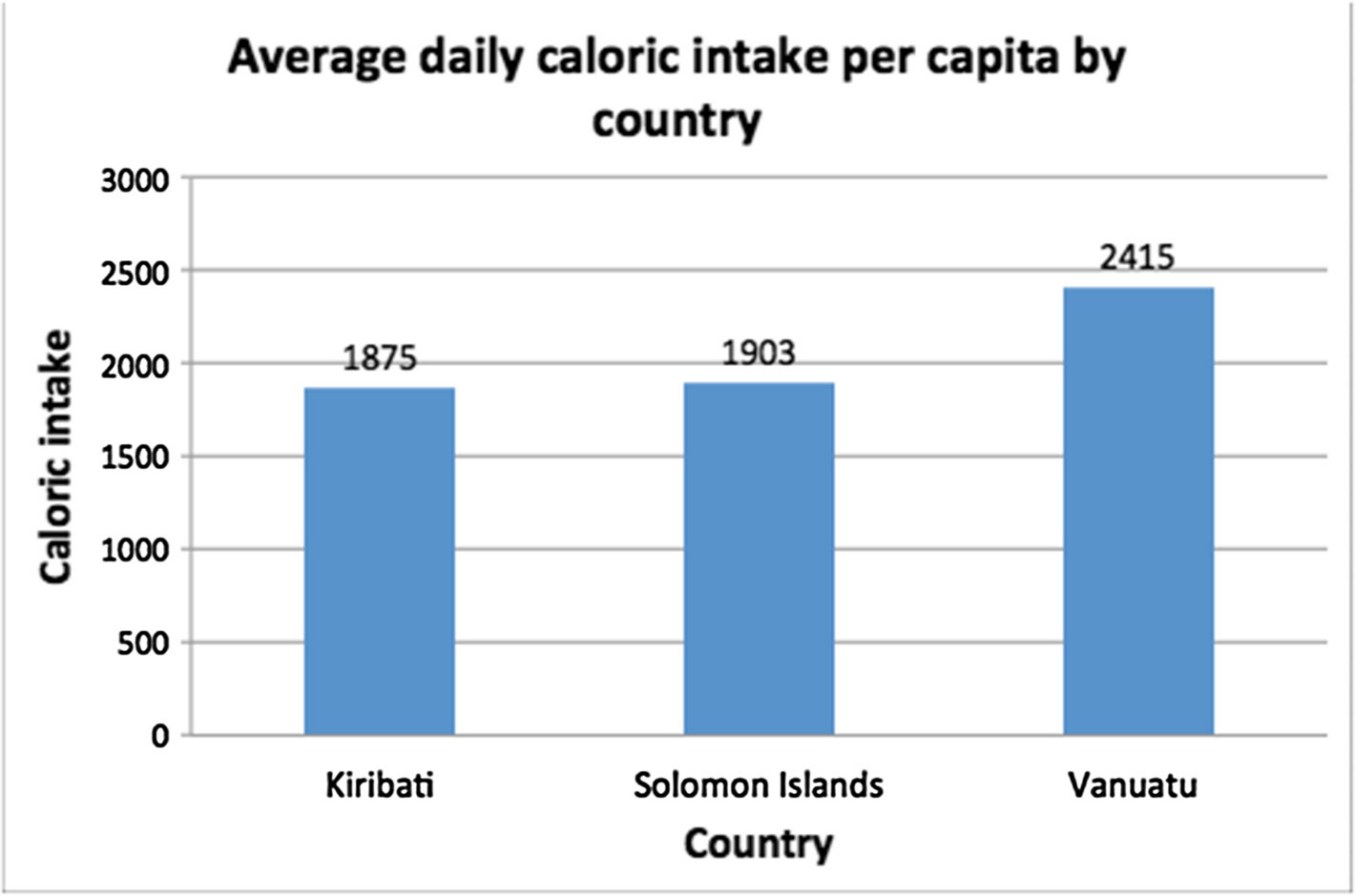
Comparison of average caloric intakes by country.

**Figure 3:**
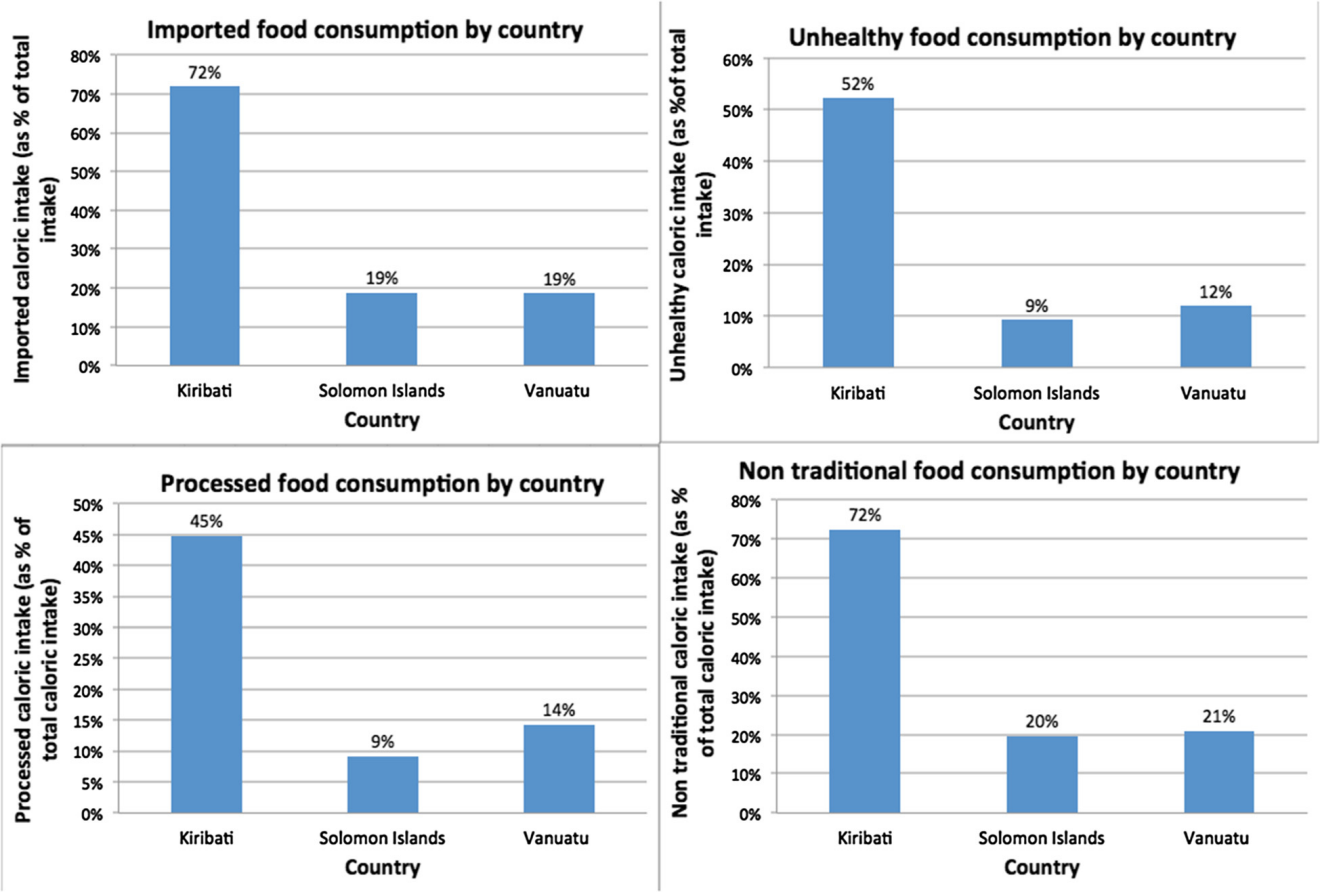
Caloric intake profiles by country.

**Figure 4:**
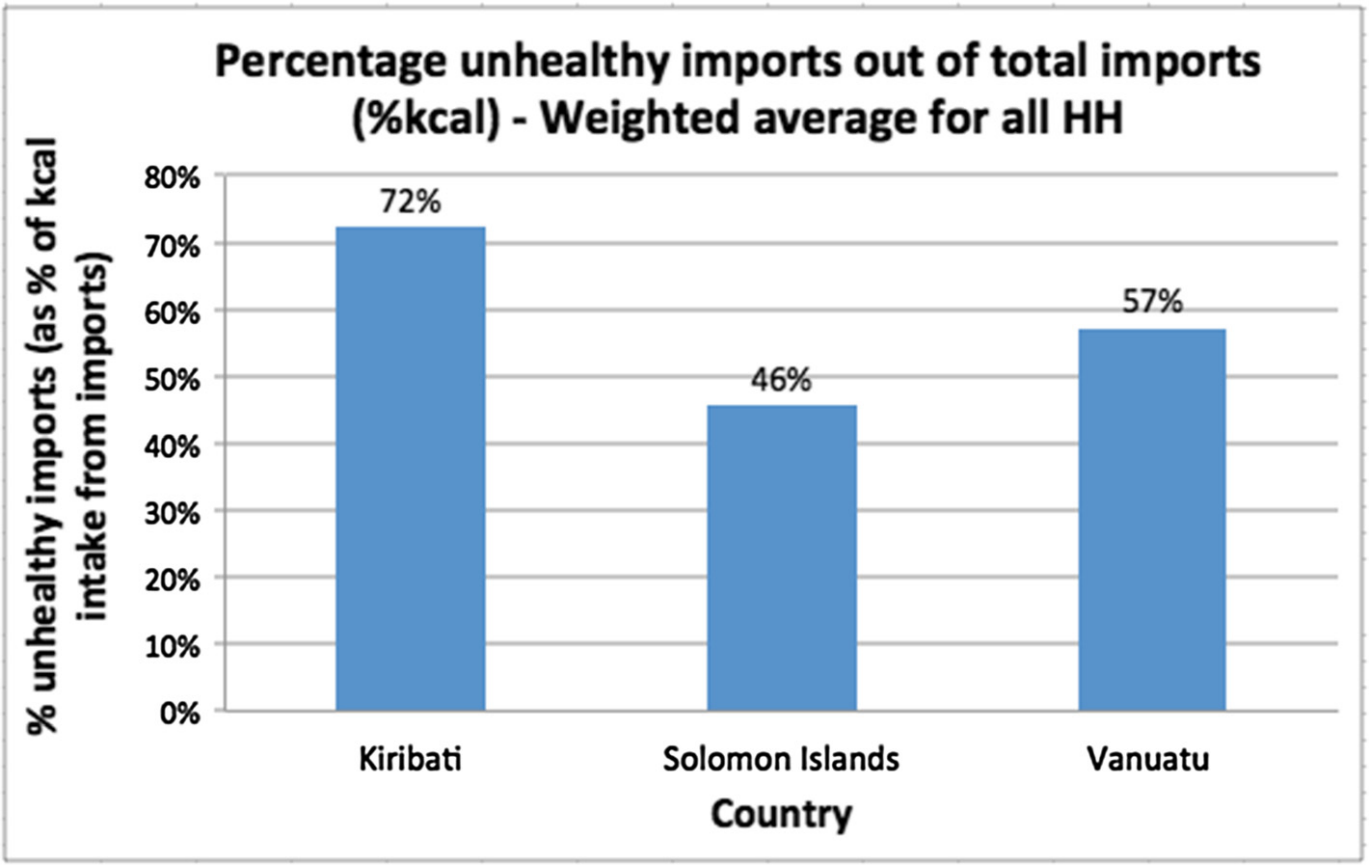
Comparison of ‘unhealthy’, imported foods.

Caloric intake profiles for the three countries were created by comparing caloric intake on each of the four food categories below. Total kcal pcae intakes differed among the three countries; Vanuatu had the highest rates while Solomon Islands and Kiribati had lower, similar values (Figure 2). The caloric intakes were, on average, lower than the global per capita average of 2780 kcal, though this could be due to measurement error and the exclusion of alcohol and food prepared outside the home [33]. To standardize daily kcal pcae intake, we used percentages in the analyses.Out of the three countries, Kiribati had, by far, the highest percentage of calories pcae from all four categories of imported, ‘unhealthy’, nontraditional and processed. Solomon Islands had the lowest percentage of calories pcae across these categories (Figure 3). This cross-country pattern in terms of calories was a more extreme illustration of the clustering evident in the food expenditure data. Kiribati had the highest expenditure on all of the aforementioned food categories, and Solomon Islands had the lowest. While households in all the countries consumed less ‘unhealthy’ and processed foods than imported and non traditional foods, these categories still represented substantial portions of total caloric intake. Also notable is that not all imported food items were ‘unhealthy’ (Figure 4).

**Figure 5:**
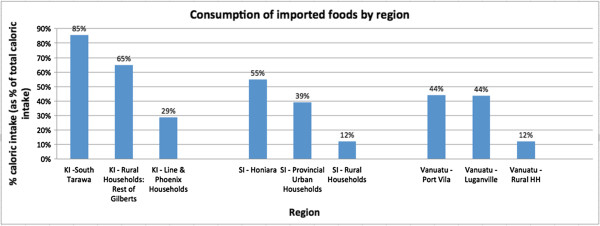
Distribution of imported food in the three countries.

The consumption of food items belonging to the four categories appeared more concentrated in urban areas in Vanuatu and Solomon Islands whereas the difference in Kiribati was between different island groups rather than between rural and urban areas, as illustrated by consumption of imported foods in Figure 5^b^.

### Breakdown of expenditure and caloric intake among imported foods

Rice accounted for the single largest expense among imported food as well as a considerable share of caloric intake from imported foods in all countries reviewed, with the exception of Tonga (Table 2). For the countries with caloric information, sugar also accounted for a large share of caloric intake from imports. The caloric intake from sugar in Kiribati was disproportionately high compared to Solomon Islands and Vanuatu, accounting for the largest percentage of any single food item in terms of total caloric intake in Kiribati (Table 2). Estimates of caloric intakes were not available for Samoa and Tonga. As a food item could be affordable but highly energy dense, and vice versa, a ranking by food expenditure alone was likely to omit food items that were more affordable but still accounted for a large share of calories. For example tinned tuna in Vanuatu accounted for 10% of food expenditure, but did not appear in the top three consumed food items in the country. The difference between expenditure and caloric intake was also clear for other items, for example the share of expenditure on sugar was less than the share of calories in Kiribati, Solomon Islands and Vanuatu (Table 2).

**Table 2:** Top 3 imported foods by country

**Country**	**Top 3 products (% of food expenditure)**	**Share of food expenditure on item out of total imported foods**	**Share of food expenditure on item out of total food expenditure**	**Top 3 products (kcal pcae/day intake)**	**Share of kcal intake per item out of total imported foods**	**Share of kcal intake per item out of total kcal intake**
Kiribati	Rice	43%	23%	Sugar	48%	34%
	Sugar	25%	13%	Rice	27%	20%
	Flour	6%	3%	Flour	11%	8%
Solomon	Rice	57%	17%	Rice	53%	10%
Islands	Noodles	13%	4%	Flour	13%	3%
	Sugar	7%	2%	Sugar	12%	2%
Vanuatu	Rice	40%	12%	Rice	34%	6%
	Tinned tuna	10%	3%	Sugar	22%	4%
	Bread	7%	2%	Bread	12%	2%
Samoa	Rice	27%	11%	n/a	n/a	n/a
	Margarine	20%	8%	n/a	n/a	n/a
	Bread and Noodles	10%	4%	n/a	n/a	n/a
Tonga	Mutton	19%	10%	n/a	n/a	n/a
	Poultry	12%	6%	n/a	n/a	n/a
	Cooked meat	10%	5%	n/a	n/a	n/a

In Tonga, mutton was the single largest expense among imported foods. Expenditure patterns, however, may not necessarily reflect similar calorie patterns. Mutton flaps, and other fatty meats, have been identified as a significant contributor to rising NCD rates in the Pacific Islands [34], but as the share of mutton flaps in the data was unknown, no inference on the impact of mutton flaps on NCDs in Tonga could be drawn from these particular data.

### Regression analysis

**Figure 6:**
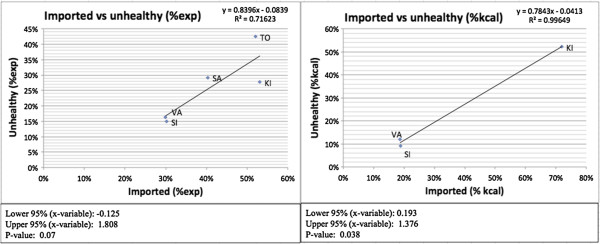
Association between imported and ‘unhealthy’ foods.

**Figure 7:**
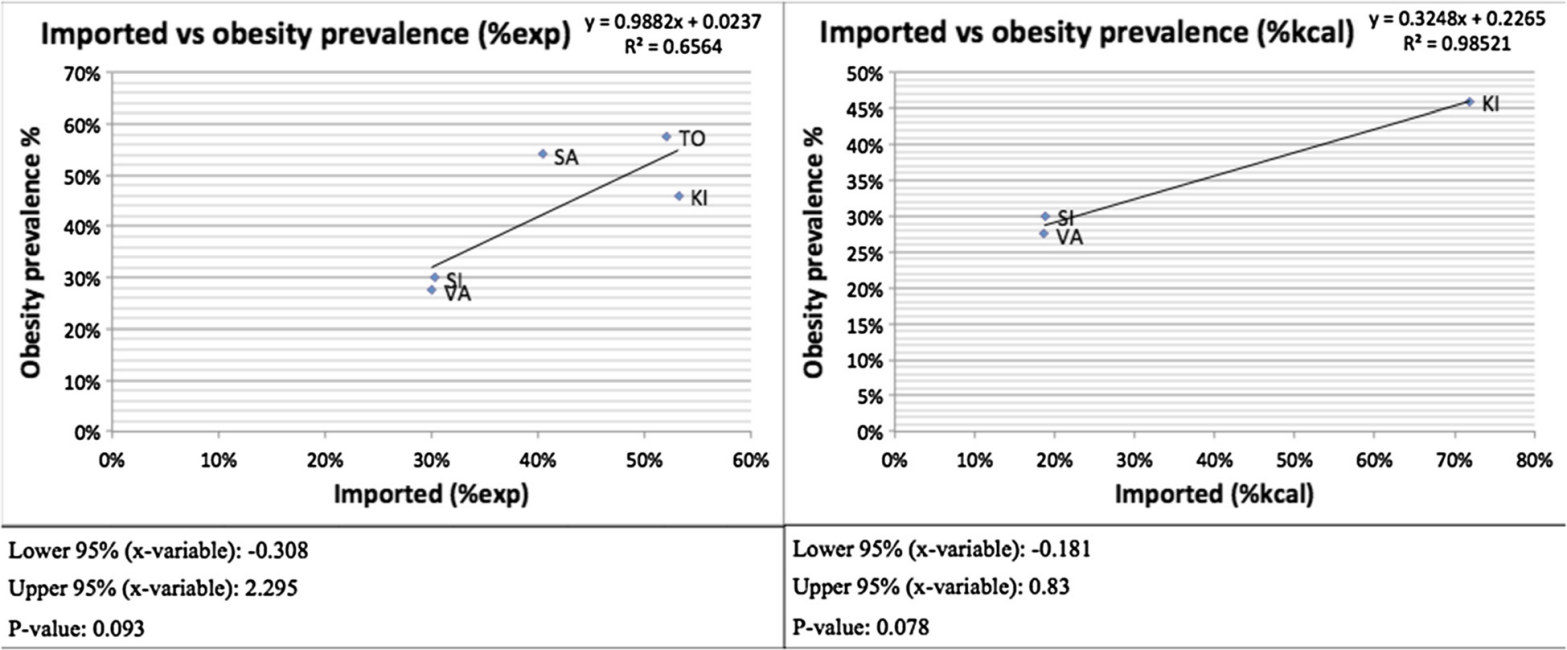
Association between imported foods and obesity prevalence.

A regression analysis found positive, statistically significant associations between the levels of imported foods and levels of ‘unhealthy’ foods when examining percentages of daily caloric intake (p = 0.038). When examining percentage food expenditure, the relationship was seemingly positive, but it was not significant at the 5% level although it was significant at the 10% level (p = 0.07). In other words, higher percentages of imports were associated with higher percentages of ‘unhealthy’ food, in terms of caloric consumption and most likely also in terms of food expenditure (Figure 6). Solomon Islands households, for example, consumed the lowest shares of imported food and ‘unhealthy’ food whereas the other countries spent and consumed more imported and ‘unhealthy’ foods. A closer look at food expenditure in Tonga and Kiribati shows that higher shares of imports are not necessarily associated with higher shares of ‘unhealthy’ foods (Figure 6). Kiribati actually had a lower share of expenditure on ‘unhealthy’ foods than Tonga while the share of expenditure on imports was slightly higher in Kiribati.There was also a positive association between imported foods and obesity prevalence. While the association was not significant at the 5% level, it was significant at the 10% level (% food expenditure: p = 0.093; % kcal intake: p = 0.078) (Figure 7). Solomon Islands and Vanuatu both consumed less imported foods and had lower obesity prevalence than did the other countries consuming more imported foods.

## Discussion

Though constrained by only a few data points in this particular analysis, the association we found between imported foods and ‘unhealthy’ foods supports previous research that shows a relationship between trade liberalization and increased intake of nutrient-poor and calorie-rich foods. Trade has been shown to affect not only diets but also obesity levels and NCD prevalence [34]. The association between trade, diet, obesity and NCD prevalence has been observed previously through case studies and has been especially well documented in some PICs such as Fiji, Tonga and Federal States of Micronesia [34-36]. While we found that, on average, imported food was significantly or near significantly associated with both ‘unhealthy’ food and obesity at a population level, some variation existed. This was especially the case with food expenditure data, where more data were available and R^2^ values were generally lower than for calorie data. For example, Tonga and Kiribati had similar shares of imported food expenditure but quite different levels of ‘unhealthy’ food expenditure (Figure 6). In fact, Kiribati had lower shares of ‘unhealthy’ food expenditure and obesity prevalence than Samoa despite having had a higher share of imports. This variation could be explained by a number of factors, including differences in trade and public health policies, which if the case, suggests that policy space may exist to mitigate the dietary harms that trade could portend. In other words, the trade-off between trade and healthy diets may not need to be as great as it would seem provided that health-sensitive policies are put in place. Urbanization has also been suggested to affect diet and accelerate nutrition transitions [37].

We did not find a consistent relationship between urban environments and consumption of imported foods in Kiribati, Solomon Islands and Vanuatu. The countries’ expenditure and caloric intake profiles appeared to differ, as did the distribution of imported foods across rural and urban areas. The apparent inconsistency across countries could possibly be explained by both differences in household distribution and differential accessibility to food items. In Kiribati, 44% [38] of the population lived in urban areas; the percentages were lower in Solomon Islands (19%) [39] and Vanuatu (26%) [40]. In Vanuatu and Solomon Islands, people in rural areas consumed less imported foods, while in Kiribati, rural households consumed relatively more imported foods. This could suggest that urbanization and food consumption are not strongly associated in these contexts or that other factors unaccounted for in our analyses, such as those affecting access, may more strongly influence the association.

The methodology had obvious limitations. One of the several sources of potential bias in the analysis was that our classification of imported and ‘unhealthy’ foods may not have been done completely independently of the other. Given that obesity prevalence was measured independently of our classification of the food data, however, the regression analysis between imports and obesity lent support to the validity of our classifications and analyses elsewhere. The classification of food items was based on a variety of simplifying assumptions, especially when classifying food as ‘unhealthy’ or ‘healthy’ (classifying an item as imported or locally produced is likely more independently verifiable). An interaction effect between imported/local foods and ‘unhealthy’/’healthy’ foods is possible (i.e., the imported share of a food item is more unhealthy than the local share). Our classification of certain products as both ‘healthy and unhealthy’ undoubtedly led to an undetermined amount of misclassification bias, which could lead to either an over- or under-estimate of the effect in our regression analyses. This simplifying assumption in particular highlights the need for HCES data to integrate stronger dietary components that allow for a more accurate assessment of nutritional profiles. Though they present challenges in our particular study, the many assumptions made – and the transparency with which they are made – also present opportunities for in-country users to engage in cross-sectoral dialogue to improve them. Users can alter classifications and assumptions accordingly in the accompanying tool.

Underreporting bias is also possible, as the expenditure diaries may not have accounted for all food expenditure. Most HIES reports used for this study did not report data on food consumption outside the home. Where reports did include consumption outside the home, we excluded such data to ensure consistency in our cross-country analysis. In addition, respondents may have underreported stigmatizing behaviors, such as the consumption of alcohol or certain unhealthy foods. Conversely, respondents may have exaggerated their consumption of healthy foods if these are believed to be socially accepted. These reinforcing sources of bias would underestimate the percentage of calories and expenditures on unhealthy foods and overestimate the same for healthy foods. Underreporting bias might also affect the rankings of unhealthy imported foods in Table 2. We do not expect under- or over-reporting to affect our cross-country analyses unless the degree of bias differs across countries, which could be due to systematic differences in respondents or in survey administration. Overall, while potential for bias exists, especially underreporting bias, some experts believe that the overall risk is low and that HIES survey results are relatively robust^c^.

Other limitations are noteworthy. Ecological analyses, as used in this study, cannot be used to infer associations between imports and NCD risk factors at an individual level [41]. Moreover, the sample size and available data did not allow for the control of potential confounders. These could be genetic, developmental (i.e., higher imports associated with higher per capita incomes and other diet and lifestyle factors) and sociocultural in nature. The populations in the five countries have different ethnic backgrounds. Polynesians have been suggested to be more susceptible to obesity while being relatively more muscular than other ethnic groups, possibly confounding the results in this analysis [42]. The observed association between obesity and imported foods may also have been confounded by other factors associated with development, such as more sedentary lifestyles, exposure to advertising or changes in dietary patterns. One example is the Tonga data, whose sampling frame consisted of the entire population. The other four countries sampled only the lowest three expenditure deciles. As such, the results for Tonga likely introduce an additional income effect that is unaccounted for in our analysis. This income effect could contribute to the generally higher rates of food expenditures on imported, processed and ‘unhealthy’ foods in Tonga. In addition, the small sample size of three and five countries may have also constrained statistical power. The ecological design, the inability to control for confounding, the singular focus on obesity to the exclusion of other key metabolic and physiological NCD risk factors and the extremely small sample size means that the regression analyses conducted should be considered as merely suggestive rather than definitive when interpreting NCD risk.

These limitations were accompanied by general limitations in HCES data. Many factors should be acknowledged when considering the usefulness of household level expenditure data (e.g. HIES, HBS, LSMS etc) for policy development. Estimating actual consumption from expenditure data has been shown to be fairly accurate but discrepancies do exist, with wastage, bulk food acquisition, food intake away from home and seasonality of produce not being accounted for adequately [15,16]. Previous research also indicates that actual caloric intake is higher than the estimation based on expenditure data, especially in low-income households [43].

Though imperfect, the methodology we developed and applied offers benefits to policymakers. Unlike STEPS and some other forms of epidemiological surveillance, the data are regularly collected and may exist over longer periods. The tool’s Excel interface is easy to use and, while classification assumptions are quite simplifying, the approach offers a platform for much-needed discussion between health and trade ministries and related sectors, especially when used in conjunction with other epidemiological data. Finally, one of the most helpful elements of the methodology is that it easily highlights the top food items that are both imported and ‘unhealthy’, allowing policymakers to develop more targeted – and perhaps more effective and feasible – policy options at the intersection of trade and health. We chose obesity as an illustration, but this tool can also be used to evaluate associations between other NCD risk factors, such as those captured by STEPs (e.g. harmful use of alcohol, smoking, etc.) and imported food. In addition to the countries included in this study, at least Palau, Federated States of Micronesia and Tuvalu have HIES reports that provide sufficiently disaggregated data to replicate the methodology easily in these countries. In addition to providing additional information to country-level policymakers, expanding the analysis to other countries in the region would provide additional, much-needed data to validate and improve the results of the cross-country regression analyses. Where appropriate HCES data exist across time within the same countries, opportunities for regression analyses of panel data may appear.

While limitations should be kept in mind, they are not debilitating, especially since the methodology should be used synergistically with other epidemiological and trade data and is most useful as a catalyst for rich cross-sectoral policy dialogue. Moreover, future modifications to the tool could address some limitations and improve functionality. One option is to incorporate sensitivity analyses so that policymakers understand which assumptions and classifications are more or less critical. Another useful modification is to use the raw household-level data that underpins the aggregated reports that we used in our analysis. Using more granular household-level data provides three important benefits. First, such data would help us understand whether an overall diet is ‘healthy’ or not, instead of looking at individual food items somewhat artificially in isolation. An illustration of this is the example of mutton in Tonga. The regional application of the methodology found that mutton was the single largest expense among imported foods in Tonga, which could serve as an entry point for future interventions on NCDs. As mutton flaps have been shown to contribute to the rising NCD epidemic in the Pacific Islands [34,44], knowing the share of mutton flaps out of mutton would be valuable. Simple algorithms could be developed and applied across more granular data to facilitate a more realistic and accurate nutritional profile. Second, more granular data would improve and enable additional statistical analyses. A distribution of diets would be known across the population, statistical power would be improved and additional confounding variables could be included, such as household socioeconomic status. Third, a policy impact module would be a useful addition. Price elasticities for ‘unhealthy’ foods, for example, would enable impact estimation of various policy measures, such as tariffs and taxes. Such enhancements would further increase the utility of HCES data and provide more insights to policymakers as they grapple with NCDs and their inter-related drivers.

The methodology and results presented in this paper are not meant to be used in isolation as the basis of policy making at the intersection of trade and NCDs. Others forms of data and inputs are needed in developing specific policies that are effective, feasible and targeted. Nonetheless, the methodology and results here have broader implications for policy and especially policy making processes. First, the results of this analysis suggest opportunities for public health measures that target key drivers of diet-related health risks. In Kiribati, for example, reducing sugar consumption would likely be a primary policy objective, given that sugar represents such a large single source of calories. Increasing the price of sugar, for example, could be one objective that could be enabled by removing sugar from the price control list^d^ and implementing a sugar tax. A direct tax on sugar would be a variant of other, more targeted taxes on sugars that have been discussed globally for years, especially taxes on soft drinks, with Mexico being the most recent example of large-scale implementation.

A sugar tax – as well as other means to increase the price of sugar – raises important design and implementation questions. Thow et al. summarize many key implementation questions based on lessons from soft drink taxes in Fiji, Samoa, Nauru and French Polynesia [45]. A potential sugar tax in Kiribati raises three specific design questions about health-promoting taxes of foods and beverages: the kind of tax, its targets and its level. Many different kinds of taxes are possible. Two of the most common types are excise taxes and ad valorem taxes. In the case of a sugar tax, the former might be preferred to the latter, as it is linked to the actual quantity of the substance and not its price. An ad valorem tax may be difficult to manage in volatile commodity markets, where it could amplify price swings. Furthermore, an ad valorem tax can be undermined more easily where price substitution among sweetened products is possible, ultimately undermining objectives of improving health and raising revenues.

An excise tax provides a more useful platform for broader taxes that are linked to sugar, fat or sodium content across different products instead of targeting individual products, like soft drinks, in isolation. Taxing individual products rather than harmful ingredients across products creates opportunities to substitute one sweetened, fatty or salty product for another. For example, while this study would suggest that Tonga and Samoa should focus on mutton and margarine, respectively, it may be more useful to consider a focus on fatty products more generally. A more general tax on fat content would include mutton and margarine as well as other high fat foods, helping to prevent substitution among fatty foods. Similarly, a tax on imported raw sugar in Kiribati might prove useful but it should not act in isolation from other products where sugar is one ingredient and that provide additional sources of dietary sugar. Properly administrating a broader excise tax on food and beverage ingredients rather than the food or beverage items themselves, comes with obvious challenges, especially in resource-constrained environments.

Finally, the level of a sugar tax in Kiribati might need to be relatively small. The fact that sugar represents a large share of food expenditure suggests that consumers may be sensitive to even small price increases. On the other hand, increasing the price of sugar may not be as feasible from a political perspective for the same reason, especially if sugar is purchased and consumed by politically powerful constituencies. An empirically-determined price elasticity of demand would be a key first step before designing a sugar tax. Understanding the price of healthier alternatives is another. The price of these alternatives could provide an index for the tax so that healthy options become more affordable than unhealthy ones. A tax on soft drinks in Samoa, for example, reportedly helped make bottled water cheaper than soft drinks [45]. A sugar tax is only one policy option among many. The suite of policy options is quite broad, including education and health promotion activities, to shape the relative availability, affordability and convenience of various foods and beverages. Many of these are outlined in WHO’s Best Buys [46]. Ultimately, the objective of these and other measures is to transform the food culture. The data presented in this study can help identify what food items to focus on and what the possible benefits and risks are of different policy approaches to aid in that transformation.

The second policy implication of this study – and of much other research that examines the impact of trade on health – is that public health policy cannot be conducted in isolation from trade policy. Many of the unhealthy foods in PICs are imported, and various trade agreements, including those agreed to by countries in acceding to the WTO, can influence public health policy space. For example, trade agreements often shape what tariff and nontariff barriers may be permissible and on what products or product categories. The agreements may also influence subsidies and other incentives that countries may want to use to promote the production and consumption of healthier foods. Furthermore, trade agreements can influence tax policy as well as rules on food labeling and advertising. Public health officials are not necessarily aware of what is legally permissible. In some cases, this can lead to an underestimate of flexibilities put in place to preserve national priorities. The WTO, for example, permits a wide range of measures to protect public health and the environment as long as measures are nondiscriminatory (i.e., they do not favor a domestic producer or one trading partner over others). Clearly understanding these rules, especially when faced with threats of legal action, is important for NCD-related policy that deals with imported foods.

Third, trade policy needs to be health-sensitive such that public health policy space is well protected. The risk is high that trade agreements are negotiated predominately through an economic lens and not necessarily through a broader human development lens, including understanding the potential negative impacts on health. Health-sensitive trade policies should not restrict policy levers whose purpose is the promotion of public health. At the very least, trade policies should provide explicit carve-outs for key NCD risk factors, such as tobacco and alcohol, as well as other food items, such as ultra-processed foods, based on national contexts. Trade policies should not only avoid restricting public health flexibilities; they should explicitly protect them in order to remove ambiguities that can inadvertently and indirectly constrain public health policy.

Fourth, cross-sectoral governance structures are required in order to enable the aforementioned recommendations. Trade and health ministries need to collaborate more systematically, especially when trade agreements are being negotiated. Even where trade and health ministries want to work together, cross-sectoral engagement can be impeded by a lack of a common language, mutual understanding and a shared agenda. Where cross-sectoral structures are evident in PICs, they may not meet regularly enough or are not sufficiently endorsed at higher political levels in order to make them effective. They would also benefit by being more inclusive. For example, civil society representation could be strengthened in order to give health and consumer groups greater voice in decision making.

Fifth and finally, south-south exchange and triangular cooperation are important. While PICs may face different specific problems, they are all facing and responding to an NCD epidemic that is being driven, at least, in part by a food environment that has been shaped by trade. Some countries have experimented with bans; others with a variety of tariffs and taxes. Given that countries are also part of overlapping trade agreements, there is an opportunity to learn from each other about what works, what does not and why. Given the economic integration within PICs in particular and the similar public health challenges that they face, PICs have an opportunity to collaborate closely on trade agreements within the region to ensure that they are sensitive to shared health challenges, including NCDs. Lessons from higher-income countries, which have struggled with NCD epidemics for years, create opportunities for renewed north-south exchange and triangular cooperation. Indeed, global coalitions across the North and South will likely be required in the face of rapidly globalizing NCD epidemics and the role of transnational companies in the epidemics’ spread. The WHO Framework Convention on Tobacco Control, a legally binding, global public health treaty, provides an example of what is possible through global cooperation in the face of daunting health challenges. Indeed, NCDs – like other global challenges, such as climate change – create opportunities to shape a new global partnership for development in a post-2015 development agenda and provide a clear focus and results-orientation for that partnership.

## Conclusion

This study supports previous research and prevailing hypotheses about the intersection of trade, diets and NCDs in the Pacific. It also provides a new, replicable and cost-effective way to utilize existing household poverty data that are regularly and systematically collected. Although food consumption data obtained through HCES surveys are no substitute for dietary survey data, such data nonetheless provide a pragmatic alternative source of valuable information for policy-relevant evidence at the nexus of trade and NCDs when precise and comparable dietary survey data are not available. It is a complement to data obtained through epidemiological and risk factor surveillance, such as those obtained through STEPs. We believe that the study can be expanded to other regions with similar issues. The accompanying tool is user friendly and suitable for use in any low- and middle-income country with existing HCES data.

## Endnotes

^a^Discussion with Wendy Snowdon on 21 December 2012.

^b^South Tarawa is a part of the Gilberts island group (Kiribati). Compare differences between KI – Rural HH: Gilberts/KI – South Tarawa and SI- Rural HH/ SI – urban HH (incl. Honiara) and VA – Rural HH/VA – urban HH (incl. Luganville and Port Vila).

^c^Discussion with David Abbott who has over 25 years of experience in Poverty Analysis in the PICs.

^d^Sugar is listed as a commodity under a price schedule according to the Ministry of Commerce, Industry and Cooperatives, Kiribati. http://www.mcic.gov.ki/?page_id=171 Accessed 5 February 2014.

## Abbreviations

FAO: Food and Agriculture Organization of the UN; FBS: Food Balance Sheets; GDP: Gross Domestic Product; HBS: Household Budget Survey; HCES: Household Consumption and Expenditure survey; HIES: Household Income and Expenditure survey; KCAL: Kilocalorie; LSMS: Living Standards Measurement Survey; NCD: Noncommunicable disease; PCAE: Per Capita Adult Equivalent; PIC: Pacific Island Country; PICTA: Pacific Island Countries Trade Agreement; PPS: Probability proportional to size; STEPs: STEPWise approach to surveillance; WHO: World Health Organization; WTO: World Trade Organization.

## Competing interests

The authors declare that they have no competing interests.

## Authors’ contributions

FS provided the initial idea to examine HIES reports in PICs in preparation for workshop proceedings, facilitated access to the reports, provided insights in Pacific islands culture and economies and contributed to editing. BL and MSE designed the methodology jointly. MSE carried out the initial drafting of both the excel tool and the manuscript. All authors edited the manuscript following reviewers comment. BL supervised and guided all processes, drafted the text on policy implications and conducted overall editing. All authors read and approved the final manuscript.
